# A prospective cohort study of long-term cognitive changes in older Medicare beneficiaries

**DOI:** 10.1186/1471-2458-11-710

**Published:** 2011-09-20

**Authors:** Fredric D Wolinsky, Suzanne E Bentler, Jason Hockenberry, Michael P Jones, Paula A Weigel, Brian Kaskie, Robert B Wallace

**Affiliations:** 1 Department of Health Management and Policy, College of Public Health, the University of Iowa, Iowa City, Iowa, USA; 2 Department of Internal Medicine, Carver College of Medicine, the University of Iowa, Iowa City, Iowa USA; 3 Department of Adult Nursing, College of Nursing, the University of Iowa, Iowa City, Iowa USA; 4 Comprehensive Access and Delivery Evaluation and Research Center (CADRE), Iowa City Veterans Administration Medical Center, Iowa City, Iowa, USA; 5 Department of Biostatistics, College of Public Health, the University of Iowa, Iowa City, Iowa, USA; 6 Department of Epidemiology, College of Public Health, the University of Iowa, Iowa City, Iowa, USA

## Abstract

**Background:**

Promoting cognitive health and preventing its decline are longstanding public health goals, but long-term changes in cognitive function are not well-documented. Therefore, we first examined long-term changes in cognitive function among older Medicare beneficiaries in the Survey on Assets and Health Dynamics among the Oldest Old (AHEAD), and then we identified the risk factors associated with those changes in cognitive function.

**Methods:**

We conducted a secondary analysis of a prospective, population-based cohort using baseline (1993-1994) interview data linked to 1993-2007 Medicare claims to examine cognitive function at the final follow-up interview which occurred between 1995-1996 and 2006-2007. Besides traditional risk factors (i.e., aging, age, race, and education) and adjustment for baseline cognitive function, we considered the reason for censoring (entrance into managed care or death), and post-baseline continuity of care and major health shocks (hospital episodes). Residual change score multiple linear regression analysis was used to predict cognitive function at the final follow-up using data from telephone interviews among 3,021 to 4,251 (sample size varied by cognitive outcome) baseline community-dwelling self-respondents that were ≥ 70 years old, not in managed Medicare, and had at least one follow-up interview as self-respondents. Cognitive function was assessed using the 7-item Telephone Interview for Cognitive Status (TICS-7; general mental status), and the 10-item immediate and delayed (episodic memory) word recall tests.

**Results:**

Mean changes in the number of correct responses on the TICS-7, and 10-item immediate and delayed word recall tests were -0.33, -0.75, and -0.78, with 43.6%, 54.9%, and 52.3% declining and 25.4%, 20.8%, and 22.9% unchanged. The main and most consistent risks for declining cognitive function were the baseline values of cognitive function (reflecting substantial regression to the mean), aging (a strong linear pattern of increased decline associated with greater aging, but with diminishing marginal returns), older age at baseline, dying before the end of the study period, lower education, and minority status.

**Conclusions:**

In addition to aging, age, minority status, and low education, substantial and differential risks for cognitive change were associated with sooner vs. later subsequent death that help to clarify the terminal drop hypothesis. No readily modifiable protective factors were identified.

## Background

The major independence threat for older adults is the loss of physical and/or mental function [1], with the latter being the more dreaded. As Rowe and Kahn noted in their classic volume [[1] p. 126]:

"Fears of cognitive loss, and especially of Alzheimer's disease, are widespread among older people. And those fears are understandable. Alzheimer's places great burdens on both the patient and those who care for them."

Although only 11% of older men and 16% of older women develop Alzheimer's disease [2], many cognitive functioning processes slow significantly with aging [3]. Accordingly, a major goal of American health policy is improving cognitive function and preventing cognitive decline [4]. Therefore, identifying all major, independent risks for changes in cognitive function is critical.

Numerous studies have focused on predicting cognitive change [5-12]. Typically, they report that the major risks for short-term (usually two- to four-year) declines have been age, aging, race, and education. Older individuals, minorities, and the less educated consistently have lower values on cognitive assessments in cross-sectional analyses, but the effects of aging, age, race, and education on long-term changes in cognitive function are mixed. Moreover, because most longitudinal studies have used homogenous (i.e., fixed) follow-up periods that are the same for all participants it has been difficult if not impossible to determine aging effects and whether or not they are nonlinear.

Accordingly, in this study we had two objectives. The first was to examine long-term (an average of 7.2 years between assessments) changes in cognitive function in a nationally representative sample of older Medicare beneficiaries in the United States. The second objective was to identify the risk factors associated with those changes in cognitive function. For both objectives we used the baseline (1993-1994) and biennial follow-up interviews through 2006 that were conducted as part of the Survey on Assets and Health Dynamics among the Oldest Old (AHEAD). Those data were then linked to Medicare claims for calendar years 1993-2007. Cognitive function was assessed using three measures: a seven item version of the Telephone Interview for Cognitive Status (TICS-7), and 10-item immediate and delayed word recall tests. Residual change score multiple linear regression was used to predict cognitive function at the final follow-up adjusted for baseline cognitive function, as well as the exposure period (aging), age, race, education, reasons for censoring, continuity of care, health shocks (post-baseline hospitalizations), and other factors.

## Methods

### Study Cohort and Sample Selection

Complete documentation of the design, procedures, and protocols for the AHEAD cohort is available online at http://hrsonline.isr.umich.edu or elsewhere [13]. We used weighted analyses to adjust for the unequal probabilities of selection that over-sampling African Americans, Hispanics, and Floridians created. An 80.4% response rate was obtained for the baseline (1993-1994) interviews, yielding 7,447 participants ≥ 70 years old (i.e., the 775 spouses < 70 years old were excluded).

An aggressive approach to maximize the completion of the biennial follow-up intervals was used (see http://hrsonline.isr.umich.edu/sitedocs/sampleresponse.pdf for specific details, including the number of participants eligible, re-interviewed, or lost to follow-up at each stage of the study). Basically, at each biennial follow-up, multiple attempts were made to re-interview all surviving participants, preferably as self-respondents, although proxy-interviews were allowed (proxy-respondent rates rose from 10.6% at baseline to 17.2% by 2006). This included re-interviews with participants who were in a nursing home at the time of the scheduled follow-up interview. The only exception to this aggressive follow-up protocol was for the 269 participants who at some point between 1995 and 2007 asked never to be contacted again. Among those who were lost to follow-up at any given biennial interview, an average of 5.5 re-interview attempts were made. The response rate among survivors at the biennial follow-up interviews ranged from a low of 93.8% in 1995-1996 to a high of 95.9% in 2006-2007, with 81.8% of the participants re-interviewed at every possible re-interview round for which they were eligible.

We limited the analytic sample to a maximum of 4,251 participants (56.5% of the total age-eligible cohort). First, we excluded 523 (7.0%) for whom proxies provided data at baseline because they were not asked the same cognitive status measures, and thus changes in cognitive function could not be assessed for them. Second, 786 (10.6%) were excluded because their baseline interviews could not be linked to their Medicare claims, and a focal interest was the association of hospitalization patterns with changes in cognitive function. Third, 610 (8.2%) who were in managed Medicare at baseline were excluded because managed care plans have different claims reporting requirements for Part B (non-institutional) claims and it is generally recommended that claims-based analyses should be limited to beneficiaries in fee-for-service Medicare [14]. Fourth, we excluded 1,077 (14.5%) because they did not have any post-baseline follow-up interviews as self-respondents, and thus changes in cognitive function could not be assessed for them. Finally, missing data on the outcome measures further reduced the analytic samples to 3,021 for the TICS-7, to 4,251 for the immediate word recall test, and to 4,068 for the delayed word recall test, reflecting higher refusal rates on the TICS-7 over time. Included participants were censored for three competing risks--death, enrolling into managed Medicare, or the 2006-2007 follow-up interviews--whichever came first.

To gauge the extent of selection bias based on these five exclusion criteria, we conducted a series of one-way analyses of variance on selected demographic (age, sex, and race), socioeconomic (education and income), and functional status (limitations in performing activities of daily living [ADLs], instrumental ADLs [IADLs], and mobility) characteristics. We constructed a categorical (i.e., nominal) measure reflecting the six possible participant groups including those excluded due to proxy-interviews at baseline, inability to link to Medicare claims, participation in managed Medicare plans, not having any follow-up interviews as a self-respondent, and refusing to answer any of the cognitive function assessments vs. being included in the analytic sample. In the Dunnett *post-hoc *test comparisons, participants included in the analytic sample were designated as the reference (or comparison) group. As expected, those results (not shown) indicated that participants in the analytic sample were younger, less likely to be a man or a member of a racial minority, and had more education, better income, and better functional status. Thus, our analytic sample was considerably advantaged relative to the members of the AHEAD cohort who were excluded from the analysis, which biases our prevalence estimates for cognitive decline downward and our estimated associated risks toward the null.

### Outcome Assessments

Cognitive function was measured using three standard assessments taken from the TICS, which originally had 11 components (TICS-11) [15]. The TICS-11 was developed for use where a standard cognitive screening test like the Mini-Mental Status Examination (MMSE) [16] could not be used, because the MMSE requires a face-to-face interview for visually presented object naming, pentagon drawing, and paper folding tasks. Thus, the MMSE and similar assessments are not usable in studies like the AHEAD that rely on telephone interviews. The reliability and validity of the TICS-11 and its comparability to the MMSE has been consistently demonstrated [17].

At baseline, AHEAD participants were asked the following questions in sequence [18]. The first was the immediate word recall test, which taps episodic verbal memory (i.e., working memory, fluid intelligence, or explicit memory). Participants were read a set of 10 words (lake, car, army, forest, ticket, bird, winter, door, mountain, and plant) and then asked to recall as many as they could over the next two minutes. Their score was the number of words (0-10) correctly recalled. The second assessment (TICS-7) asked participants to: (1) correctly identify the month, day, year, and day of the week (0-4 points); (2) count backwards from 20 (2 points if correct on the first try, 1 point if correct on the second try); (3) name a tool used to cut paper (scissors; 1 point); (4) name a prickly plant found in the desert (cactus; 1 point); (5) name the current President of the U.S. (1 point); (6) name the current Vice President of the U.S. (1 point); and, (7) serially subtract 7 from 100 five times (0-5 points). TICS-7 scores ranged from a low of 0 correct answers to a high of 15 correct answers. The third assessment was the delayed word recall test, which also taps episodic memory (i.e., acquired information, and processed and stored memory retrieval). Here again, the score was the number of words (0-10) correctly recalled over the next two minutes.

The initial psychometric reports from the AHEAD cohort [18] combined the three cognitive function assessments into a summary measure ranging from 0 to 35 (TICS-9), as did many subsequent analyses [9]. We, however, treated the TICS-7, and the immediate and delayed word recall tests as three separate measures. The reason was that both exploratory and confirmatory factor analyses have consistently shown that the TICS-7 items and the immediate and delayed word recall tests load on two separate factors, providing strong evidence of the multidimensionality of the underlying latent variable--cognitive function [18]. All of the TICS-7 items loaded principally on the first factor, which clearly reflected general mental status. In contrast, both the immediate and delayed word recall tasks loaded on a second factor that clearly reflected memory. Furthermore, the immediate word recall test measured working memory, fluid intelligence, or explicit memory, whereas the delayed word recall test was generally viewed as a measure of acquired information, and processed and stored memory retrieval, with the latter being a more demanding task [15] and a better prognostic indicator of dementia risk [17].

Post-baseline, modest changes in the three cognitive assessments occurred to minimize learning (or practice) curves associated with prior administration. The immediate and delayed word recall tests were modified in that four alternative 10-word lists were used, with random assignment to one of the alternative lists at each subsequent re-interview. Using alternative forms of word recall lists in this fashion minimizes learning (practice) curves and results in highly reliable alternative forms reliability, with coefficients ranging from 0.67 to 0.90 [19,20]. The starting number for counting backwards was also changed, and alternative objects were substituted for the scissors and cactus naming questions.

### Exposure and Censoring Adjustment

The time periods between the baseline and last follow-up interview were not uniform, with participants having 2-12 years between their baseline and last follow-up interviews. To adjust for this differential exposure (aging effects) to cognitive change we included the number of years (centred at the mean) from the baseline interview to the last follow-up completed as a self-respondent. The quadratic of the centred exposure was also included to test for nonlinearity. Exposure time could be censored based on two criteria. The first was due to entering Medicare managed care plans. To adjust for this we created a single binary indicator reflecting censoring due to entering managed care, regardless of vital status at the end of the study period (January 2008). The second criterion was vital status. To adjust for this we included a set of three binary indicators reflecting surviving until the end of the study period, dying within one year after the last completed follow-up (reflecting terminal decline) [21,22], or dying more than one year after the last completed follow-up (reflecting delayed deaths) as the reference group.

### Risk Factors

Potential risks for changes in cognitive function can be categorized into demographic and socioeconomic characteristics, disease history, health lifestyles and functional status [5-12], all of which were assessed at baseline. Although some of these characteristics, especially functional status, may have changed after baseline, we took a static approach to their assessment because the focal variables among them are generally fixed, and not all participants in the analytic sample were continuously re-interviewed as self-respondents. This approach likely credits any associations in adverse changes in these characteristics with changes in cognitive function to the aging coefficients, potentially resulting in their overestimation. To these we added post-baseline continuity of care, given its beneficial role in primary care for older adults [23], as well as post-baseline "health shocks" indexed somewhat by physician visits but mostly by hospitalizations [24,25].

Demographic and socioeconomic factors included age, sex, race, marital status, education, and income. Disease history was measured by a set of 10 binary indicators for whether the participant reported having been told by a physician that she had the particular diseases, and a binary indicator of ≥ 3 of the diseases to tap comorbidity. Body mass index, engaging in vigorous physical exercise, cigarette smoking, and alcohol consumption was used to measure health lifestyles. Functional status included measures of ADLs, IADLs, mobility, vision, hearing, and depressive symptoms [18]. We used a previously validated measure of continuity of care with a primary care physician [26-28] to calculate the percentage of days between baseline and the last follow-up interview for which continuity of care existed (no more than an 8-month interval between office visits to the same primary care physician), and converted it to two binary indicators reflecting the extremes of always or never having continuity.

Using the Medicare claims Carrier Statistical Analytic File (SAF) and standard accounting methods we summed post-baseline primary care physician visits across the observation period, and divided this sum by the number of follow-up years. This distribution was then categorized into a set of three binary indicators reflecting the lowest, second, and third vs. the highest quartiles. We measured post-baseline hospital episodes in a similar fashion, with this distribution also categorized into a set of three binary indicators reflecting the lowest (none), second, and third vs. the highest quartiles.

### Analysis

There are two common multivariable linear regression approaches to modelling changes in metric (interval level of measurement) outcomes like cognitive function [29-33]. One approach, often referred to as the "delta" approach, involves the use of change (or gain) scores, where the dependent variable is the difference (delta) between the baseline and follow-up values (i.e., time_2 _- time_1_) on the outcome of interest. The other approach, often referred to as the "residual change score" approach, involves having the follow-up value on the outcome of interest predicted by the baseline value, as well as other risk factors (or covariates). For the purpose of this study, the residual change score approach was more appropriate.

In its simplest form, the residual change score approach involves using a multivariable linear regression modelling approach that may be depicted as:

$$C_{2}=a+b_{1}C_{1}+b_{2}X_{1}+e$$

where *C *is the cognitive function measure (either the TICS-7, or immediate or delayed word recall assessments), the subscripts refer to *t_2 _*(the final follow-up) and *t_1 _*(the baseline interview), *a *is the intercept, *b_1 _*is the effect of the baseline cognitive function measure on the cognitive function measure at the final interview, *X *is a vector of other risk factors, *b_2 _*is a vector of coefficients for the *X *vector of other risk factors, and *e *is the error term. Values of *b_1_*, the effect of the baseline cognitive function measure, that are less than 1 reflect regression to the mean, whereas values greater than 1 reflect increasing inequalities (i.e., accumulative advantage and/or accumulative disadvantage), with values of 1 reflecting stability. The *b_2 _*vector directly represents the effects of the *X *vector of other risk factors on changes in cognitive function. Besides its straightforward nature and interpretation, the advantage of the residual change score regression approach in the current context was that it avoided the potential bias of producing spurious correlations of risk factors that may occur in the delta approach when the risk factors are associated with the baseline value of the outcome, which is sometimes referred to as horse-racing bias [34,35], and Lord's paradox in which the delta approach fails to identify associations evident from residual change score analysis of the same data [36-39].

Statistical assumptions about the regression models, including additivity of the effects, were evaluated using standard procedures. Given the large number of potential risk factors, we tested for multicollinearity, but found no problems (all VIFs [variance inflation factors] were below 2.9). Nonetheless, as an added safeguard stepwise and backward model trimming procedures were also used, but yielded equivalent results [40]. The majority of the missing data occurred for the dependent variables due to the use of proxy-respondents at follow-up (see study cohort and sample selection, above). The minimal amount of missing data on the risk factors (independent variables) was addressed using list-wise deletion.

### Human subject approvals

The research reported here was supported by a USA National Institutes of Health (NIH) grant (R01 AG022913) to Dr Wolinsky. The research and restricted data protection plans associated with this grant were approved by AHEAD on February 20, 2003 (#2003-006). Furthermore, the human subject protocol for this USA NIH grant was fully approved by University of Iowa Institutional Review Board (IRB-01) on March 24, 2003, and was fully approved again by IRB-01 at each of its annual reviews, the most recent of which occurred on June 7, 2011. A Data Use Agreement (DUA) with the USA Centres for Medicare and Medicaid Services (CMS; DUA 14807) for this study was fully approved on March 3, 2005. Written informed consent was obtained.

## Results

### Sample characteristics

Table 1 contains the percentages for the categorical variables and the means and standard deviations for the continuous variables in the analytic sample. Mean age was 78, 37% were men, 9% were African American, and 3% were Hispanic. About half (52%) were married and 37% widowed, with 22% having only attended grade school and 30% going to college. Seventeen per cent had ≥ 3 chronic conditions, 14% were obese, a third engaged in vigorous exercise, 9% were current smokers, and 43% were former smokers. Twenty-eight per cent were never hospitalized post-baseline, and 27% did not have continuity of care at any time. The baseline means on the outcomes were 12.9 on the TICS-7, and 4.0 and 2.6 on the immediate and delayed word recall tests.

**Table 1:** Percentages or Means and Standard Deviations of the Variables

Independent Variables	Percentage	Mean	Standard Deviation
*Baseline Cognition*			
Telephone Interview for Cognitive Status (TICS-7; 0-15 correct)		12.9	2.1
Immediate Word Recall (0-10 correct)		4.0	1.8
Delayed Word Recall (0-10 correct)		2.6	2.0
			
*Exposure and Censoring*			
Years Between Interviews		7.2	4.1
Entered Managed Care After Baseline	15.5		
Survived to the End of the Study^a^	35.1		
Died ≤ 1 Year After the Last Interview^a^	18.9		
			
*Demographic and Socioeconomic*			
Age		77.7	5.3
Men	36.9		
African American^b^	8.7		
Hispanic American^b^	3.2		
Widowed^c^	39.4		
Divorced or Separated^c^	5.0		
Never Married^c^	3.2		
Grade School Only^d^	21.6		
Attended College^d^	29.8		
Lowest Income Quintile^e^	13.1		
Highest Income Quintile^e^	26.5		
			
*Disease History and Comorbidity*			
Acute Myocardial Infarction	6.1		
Angina	7.9		
Arthritis	24.8		
Cancer	12.2		
Diabetes	10.9		
Hip Fracture	3.9		
Hypertension	44.2		
Lung Conditions	7.6		
Psychological Conditions	6.2		
Stroke	7.2		
Comorbidity (≥ 3 of the above diseases)	16.7		
*Health Lifetyles*			
Obese^f^	14.2		
Underweight^f^	2.5		
Engages in Vigorous Exercise (≥ 3 times per week)	33.0		
Current Cigarette Smoker^g^	9.4		
Former Cigarette Smoker^g^	42.6		
≥1 Alcoholic Drink Daily	11.6		
			
*Functional Status*			
Activities of Daily Living Limitations (0-5)		0.25	0.69
Instrumental ADL Limitations (0-5)		0.21	0.63
Mobility Limitations (0-4)		1.13	1.42
Good Vision	77.4		
Good Hearing	76.8		
Depressive Symptoms		1.5	1.9
			
*Continuity of Care*			
Every Day After Baseline^h^	9.2		
Never After Baseline^h^	26.7		
			
*Post-Baseline Health Shocks*			
Quartile 1 of Annual Primary Care Visits^i^	25.0		
Quartile 2 of Annual Primary Care Visits^i^	25.0		
Quartile 3 of Annual Primary Care Visits^i^	25.0		
Quartile 1 of Annual Hospital Episodes^j^	27.9		
Quartile 2 of Annual Hospital Episodes^j^	24.7		
Quartile 3 of Annual Hospital Episodes^j^	22.5		

^a^Reference category is died > 1 year after the last follow-up interview.

^b^Reference category is White.

^c^Reference category is married.

^d^Reference category is high school.

^e^Reference category is the three middle quintiles.

^f^Reference category is normal or overweight.

^g^Reference category is never having smoked cigarettes.

^h^Reference category is continuity of care for 1-99%.

^i^Reference category is top quartile of primary care visits.

^j^Reference category is top quartile of annual hospital episodes.

Follow-up data collection for 15.5% of the participants was censored due to their entrance into Medicare managed care plans at some point after baseline. About half of these individuals survived until the end of the study (January 2008). In terms of vital status, 35.1% of the participants in the analytic sample survived until the end of the study, with about one-fourth entering managed care. Among the decedents (64.9%), 29.1% died ≤ 1 year after their last follow-up interview, and 70.9% died > 1 year afterwards. The mean number of years between the baseline and last interview was 7.2 and ranged from 2-12 years with 24.3% having their last re-interview in 1995-1996, 16.8% in 1998-1999, 12.2% in 2000, 10.3% in 2002-2003, 9.9% in 2004-2005, and 26.5% in 2006-2007. Between baseline and the last follow-up interview, the mean changes in the number of correct responses on the TICS-7, and 10-item immediate and delayed word recall tests were -0.33, -0.75, and -0.78, with 43.6%, 54.9%, and 52.3% declining and 25.4%, 20.8%, and 22.9% unchanged.

### Crude cognitive changes by age, race, and education

Tables 2, 3, and 4 contain the means (and their 95 per cent confidence intervals) at baseline, the last follow-up, and the resulting change scores on the cognitive outcomes by age, race, and education categories, respectively. An uninterrupted monotonic pattern of decline was shown by age categories for the baseline and final follow-up values for all three cognitive outcomes, with all differences being statistically significant. In contrast, none of the change scores significantly varied by age category. Whites had higher baseline and final follow-up values for all three cognitive outcomes than either Hispanics or African Americans. There were no significant differences by race on changes in the TICS-7, but there were on the immediate and delayed word recall tests, where Whites had the largest declines. An uninterrupted monotonic pattern of decline was also shown by education categories for the baseline and final follow-up values for all three cognitive outcomes, with all differences being statistically significant. There were also no significant differences by education on changes in the TICS-7, but there were on the immediate and delayed word recall tests, with those having grade school educations declining the least.

**Table 2:** Mean (and 95% confidence interval) baseline, final follow-up, and change scores on the TICS-7, immediate word recall, and delayed word recall tests by age categories

Independent Variable	Ages 69-75	Ages 76-80	Ages 81-85	Ages 86 and Older
*Baseline*				
TICS-7***	13.5 (13.4, 13.6)	13.1 (12.9, 13.2)	12.9 (12.7, 13.1)	12.4 (12.0, 12.8)
Immediate Word Recall***	5.1 (5.1, 5.2)	4.6 (4.5, 4.7)	4.1 (4.0, 4.2)	3.7 (3.5, 3.9)
Delayed Word Recall***	3.9 (3.8, 4.0)	3.1 (3.0, 3.3)	2.5 (2.3, 2.6)	2.0 (1.8, 2.2)
				
*Final Follow-Up*				
TICS-7***	13.2 (13.2, 13.3)	12.7 (12.6, 12.9)	12.4 (12.2, 12.7)	11.8 (11.4, 12.2)
Immediate Word Recall***	4.4 (4.3, 4.5)	3.8 (3.7, 3.9)	3.3 (3.2, 3.5)	2.9 (2.7, 3.0)
Delayed Word Recall***	3.1 (3.0. 3.2)	2.4 (2.3, 2.5)	2.0 (1.8, 2.1)	1.5 (1.3, 2.7)
				
*Change Scores*				
TICS-7	-0.3 (-0.4, -0.2)	-0.3 (-0.5, -0.2)	-0.5 (-0.8, -0.2)	-0.6 (-1.0, -0.2)
Immediate Word Recall	-0.7 (-0.8, -0.7)	-0.8 (-0.9, -0.6)	-0.7 (-0.9, -0.6)	-0.8 (-1.0, -0.2)
Delayed Word Recall	-0.8 (-0.9, -0.7)	-0.8 (-0.9, -0.7)	-0.6 (-0.8, -0.5)	-0.7 (-0.9, -0.4)
				

* = p < 0.05; ** = p < 0.01; *** = p < 0.001

**Table 3:** Mean (and 95% confidence interval) baseline, final follow-up, and change scores on the TICS-7, immediate word recall, and delayed word recall tests by race categories

Independent Variable	Hispanic	African American	White
*Baseline*			
TICS-7***	11.9 (11.3, 12.4)	11.7 (11.2, 12.2)	13.4 (13.3, 13.4)
Immediate Word Recall***	3.8 (3.5, 4.1)	3.6 (3.4, 3.8)	4.9 (4.8, 4.9)
Delayed Word Recall***	2.3 (2.0, 2.6)	2.1 (2.0, 2.3)	3.5 (3.4, 3.6)
			
*Final Follow-Up*			
TICS-7***	11.4 (10.9, 12.0)	11.6 (11.1, 12.0)	13.0 (12.9, 13.1)
Immediate Word Recall***	3.2 (2.9, 3.4)	3.3 (3.1, 3.5)	4.1 (4.0, 4.1)
Delayed Word Recall***	2.0 (1.7, 2.3)	1.8 (1.6, 2.0)	2.8 (2.7, 2.8)
			
*Change Scores*			
TICS-7	-0.4 (-1.0, 0.2)	-0.2 (-0.6, 0.3)	-0.3 (-0.4, -0.3)
Immediate Word Recall***	-0.7 (-1.0, -0.4)	-0.3 (-0.5, -0.1)	-0.8 (-0.9, -0.7)
Delayed Word Recall***	-0.3 (-0.7, 0.6)	-0.4 (-0.7, -0.2)	-0.8 (-0.9, -0.8)

* = p < 0.05; ** = p < 0.01; *** = p < 0.001

**Table 4:** Mean (and 95% confidence interval) baseline, final follow-up, and change scores on the TICS-7, immediate word recall, and delayed word recall tests by education categories

Independent Variable	Grade School	High School	College
*Baseline*			
TICS-7***	12.1 (11.9, 12.4)	13.2 (13.1, 13.3)	13.8 (13.7, 13.9)
Immediate Word Recall***	3.7 (3.6, 3.8)	4.8 (4.7, 4.9)	5.3 (5.2, 5.4)
Delayed Word Recall***	2.2 (2.1, 2.3)	3.5 (3.4, 3.6)	3.9 (3.8, 4.0)
			
*Final Follow-Up*			
TICS-7***	11.8 (11.6, 12.1)	12.9 (12.8, 13.0)	13.4 (13.3, 13.5)
Immediate Word Recall***	3.2 (3.1, 3.3)	4.0 (3.9, 4.1)	4.5 (4.4, 4.6)
Delayed Word Recall***	1.9 (1.8, 2.0)	2.7 (2.6, 2.8)	3.1 (2.9, 3.2)
			
*Change Scores*			
TICS-7	-0.3 (-0.6, -0.3)	-0.3 (-0.4, -0.2)	-0.4 (-0.5, -0.3)
Immediate Word Recall***	-0.5 (-0.6, -0.4)	-0.8 (-0.9, -0.7)	-0.8 (-0.9, -0.7)
Delayed Word Recall***	-0.4 (-0.5, -0.3)	-0.8 (-0.9, -0.7)	-0.9 (-1.0, -0.8)

* = p < 0.05; ** = p < 0.01; *** = p < 0.001

### Multivariable linear regression models

Table 5 contains the unstandardized (*b*) and standardized (*β*) adjusted regression coefficients from the residual change score multivariable linear regression models. The *b *coefficients reflect the effect of a one unit increase in the independent variable as originally measured on the dependent variable as originally measured, whereas the *β *coefficients reflect the effect of a one standard deviation increase in the independent variable on a one standard deviation increase in the dependent variable. That is, the *b *coefficients express the effect size in the original units of measurement (i.e., what is the effect of one more year of education on changes in the number of words correctly recalled), whereas the *β *coefficients express the effect size in standard units and may be directly compared to determine relative effect size.

**Table 5:** Unstandardized (*b*) and standardized (*β*) regression coefficients from the multivariable linear regression models predicting cognitive function at the last follow-up interview

Independent Variables	TICS-7 at Follow-Up (*N *= 3,002)		Immediate Word Recall at Follow-Up (*N *= 4,211)		Delayed Word Recall at Follow-Up (*N *= 4,033)	
	*b*	*β*	*b*	*β*	*b*	*β*
*Baseline Cognition*						
Telephone Interview for Cognitive Status (TICS-7; 0-15 correct)	0.36***	0.33				
Immediate Word Recall (0-10 correct)			0.34***	0.34		
Delayed Word Recall (0-10 correct)					0.29***	0.32
*Exposure and Censoring*						
Years Between Interviews	-0.07***	-0.13	-0.05***	-0.11	-0.05***	-0.11
Years Between Interviews Squared	0.01*	0.05	0.01***	0.09	0.01***	0.08
Entered Managed Care After Baseline	0.42***	0.07	0.46***	0.09	0.50***	0.09
Survived to the End of the Study^a^	0.67***	0.14	0.33***	0.08	0.45***	0.10
Died ≤ 1 Year After the Last Interview^a^	0.22*	0.04	0.15*	0.03	0.10	0.02
*Demographic and Socioeconomic*						
Age	-0.05***	-0.11	-0.06***	-0.16	-0.06***	-0.16
Men	0.17*	0.04	-0.12	-0.03	-0.17*	-0.04
African American^b^	-0.71***	-0.07	-0.20*	-0.03	-0.44***	-0.06
Hispanic American^b^	-0.57*	-0.04	-0.20	-0.02	-0.12	-0.01
Widowed^c^	-0.08	-0.02	-0.01	-0.00	-0.03	-0.01
Divorced or Separated^c^	0.10	0.01	0.05	0.01	0.29*	0.03
Never Married^c^	0.30	0.03	0.21	0.02	-0.02	-0.00
Grade School Only^d^	-0.43***	-0.07	-0.26***	-0.06	-0.17*	-0.03
Attended College^d^	0.20**	0.04	0.27***	0.07	0.25***	0.06
Lowest Income Quintile^e^	-0.21	-0.03	-0.03	-0.01	-0.12	-0.02
Highest Income Quintile^e^	0.17	0.04	-0.02	-0.00	0.02	0.01
*Disease History and Comorbidity*						
Acute Myocardial Infarction	0.11	0.01	0.12	0.02	0.07	0.01
Angina	-0.14	-0.02	0.04	0.01	-0.06	-0.01
Arthritis	-0.28**	-0.06	-0.06	-0.02	0.07	0.02
Cancer	0.04	0.01	0.11	0.02	0.15	0.03
Diabetes	-0.08	-0.01	-0.15	-0.03	-0.21*	-0.03
Hip Fracture	-0.10	-0.01	-0.01	-0.00	0.25	0.03
Hypertension	0.10	0.02	0.05	0.01	0.07	0.02
Lung Conditions	0.07	0.01	0.06	0.01	-0.02	-0.00
Psychological Conditions	-0.21	-0.02	-0.21	-0.03	-0.05	-0.01
Stroke	-0.13	-0.02	-0.14	-0.02	-0.30*	-0.04
Comorbidity (≥ 3 of the above diseases)	0.24	0.04	0.19*	0.04	0.26*	0.05
*Health Lifetyles*						
Obese^f^	0.16	0.03	0.08	0.02	-0.01	-0.00
Underweight^f^	0.02	0.00	0.09	0.01	-0.05	-0.00
Engages in Vigorous Activity (≥ 3 times per week)	-0.08	-0.02	0.04	0.01	-0.01	-0.00
Current Cigarette Smoker^g^	-0.02	-0.00	-0.15	-0.02	-0.13	-0.02
Former Cigarette Smoker^g^	0.11	0.03	0.01	0.00	0.02	0.01
≥ 1 Alcoholic Drink Daily	0.02	0.00	-0.02	-0.00	-0.02	-0.00
*Functional Status*						
Activities of Daily Living Limitations	-0.06	-0.02	-0.06	-0.02	-0.03	-0.01
Instrumental ADL Limitations	-0.07	0.02	-0.11*	-0.04	-0.08	-0.02
Mobility Limitations	0.08*	-0.05	0.03	0.02	0.00	0.00
Good Vision	-0.01	-0.00	-0.07	0.02	-0.01	-0.00
Good Hearing	-0.14	-0.03	0.13*	0.03	-0.02	-0.00
Depressive Symptoms	-0.03	-0.02	-0.04*	-0.04	-0.02	-0.02
*Continuity of Care*						
Every Day After Baseline^h^	0.17	0.02	0.02	0.04	0.09	0.01
Never After Baseline^h^	-0.22	-0.05	-0.03	-0.01	0.03	0.01
*Post-Baseline Health Shocks*						
Quartile 1 of Annual Primary Care Visits^i^	0.43**	0.09	0.01	0.00	0.12	0.03
Quartile 2 of Annual Primary Care Visits^i^	0.38***	0.08	0.01	0.00	0.16	0.04
Quartile 3 of Annual Primary Care Visits^i^	0.13	0.03	-0.01	-0.00	0.10	0.02
Quartile 1 of Annual Hospital Episodes^j^	0.09	0.02	0.16*	0.04	0.08	0.02
Quartile 2 of Annual Hospital Episodes^j^	0.01	0.00	-0.01	-0.00	-0.07	-0.02
Quartile 3 of Annual Hospital Episodes^j^	0.28**	0.06	-0.04	-0.00	-0.07	-0.02
						
Intercept	10.97***		6.30***		5.89***	
R^2^	0.24***		0.28***		0.25***	

* = p < 0.05; ** = p < 0.01; *** = p < 0.001

^a^Reference category is died > 1 year after the last follow-up interview.

^b^Reference category is White.

^c^Reference category is married.

^d^Reference category is high school.

^e^Reference category is the three middle quintiles.

^f^Reference category is normal or overweight.

^g^Reference category is never having smoked cigarettes.

^h^Reference category is continuity of care for 1-99%.

^i^Reference category is top quartile of primary care visits.

^j^Reference category is top quartile of annual hospital episodes.

In general, the main and most consistent risks for cognitive change, in descending order based on the *β *coefficients, were the baseline value of cognitive function, aging (the combination of the linear and nonlinear terms), age, surviving to the end of the study or being censored due to entrance into managed care plans, education (the combination of the grade school and college terms), and race. The effect of the baseline cognitive function measures

(*b *< 1.00) indicated substantial regression to the mean. More aging (years between interviews) was independently associated with greater declines in cognitive status, with the declines associated with aging being nonlinear and diminishing at the margin. Older age at the time of the baseline interview was also associated with greater declines in cognitive status. Participants who either survived to the end of the study or entered managed care had significantly less cognitive decline than those who died, although decedents who died within one year after their last follow-up declined less than those who lived longer before dying. Participants with grade school educations declined more than those going on to high school, while those attending college had the smallest declines in cognitive status. African Americans had greater cognitive decline than Whites on all three outcomes, while Hispanics had greater decline than Whites but only on the TICS-7.

## Discussion

We examined long-term changes in three standard cognitive outcome measures--the TICS-7 (general mental status), and immediate and delayed 10-word recall (episodic verbal memory) tests. Data included the baseline (1993-1994) and the last biennial follow-up interviews through 2006 from AHEAD self-respondents linked to their Medicare claims for 1993-2007. To predict cognitive function at the last follow-up interview, residual change score multiple linear regression analysis was used to adjust for baseline cognitive status, exposure period, reasons for censoring, age, race, education, and other factors.

We found that the major and consistent risks for cognitive decline were the baseline values of cognitive function, aging, age, surviving to the end of the study or being censored due to entrance into managed care plans, education, and race. There was substantial regression to the mean for all three cognitive function tests. Having aged longer (i.e., more years had occurred between the baseline and last follow-up interviews) and being older at baseline were independently associated with greater declines in cognitive status. The aging effect was nonlinear reflecting diminishing returns at the margins (i.e., when participants were followed for longer periods of time). Decedents had more decline in cognitive function than those who survived until the last biennial interview or those who were censored because they entered managed care; but among decedents, those who died within one year of their last follow-up interview declined less than those who lived longer before dying. Those with grade school educations declined more than those going on to high school, with participants who attended college having the smallest declines. African Americans had larger cognitive declines than Whites on all outcomes, while Hispanics had greater declines on the TICS-7 than Whites. With some interesting exceptions, these findings are generally consistent with previous reports in the literature [41-45], although it is worth noting that a recent USA NIH Conference systematic review led the select panel to conclude that:

"...among the extensive list of putative risk or protective factors we reviewed, few had sufficient evidence from which to draw firm conclusions about their association with cognitive decline" [46].

The exceptions to the general consistency of our results with the extant literature involve one association with declines in cognitive function that we did find, and four that we did not.

First, we did observe a decedent effect. The general decedent effect was that AHEAD participants who died before the end of the observation period had greater declines in cognitive function than those who survived, or than those who left fee-for-service Medicare for Medicare managed care plans. However, we also found that there was greater observed cognitive decline among decedents who lived at least one year beyond their last follow-up interview vs. decedents who died within a year of their final follow-up interview. This was not expected, and suggests that the longstanding terminal drop hypothesis needs clarification. That hypothesis states that the greatest declines in cognitive function should occur during a short-period prior to death [21,23]. In contrast, we found that while cognitive declines were associated with pending mortality, when death was further off in the horizon, greater cognitive declines occurred. We suspect that either (a) participants who lived longer after their last follow-up interview before dying were more likely to become demented and suffer, along with their caregivers, the prolonged agony of Alzheimer's disease [1], or that (b) participants who died within one year of their final follow-up interview encountered more sudden or compressed morbidity changes that were not adequately captured by our study design. In either case, further research is needed to evaluate these possibilities.

The first association with declines in cognitive functioning that we did not find involved health shocks indexed by post-baseline hospitalizations. This contradicts recent evidence that hospitalizations, especially for critical illness, lead to cognitive decline in older adults [46]. There are two plausible explanations for this discrepancy. On the one hand, we focused on the volume rather than intensity of post-baseline hospitalizations, and we used an indicator averaged over the entire follow-up period. The implicit assumption of this approach is that hospital episodes are uniformly distributed across the life course, which may be erroneous. Treating hospitalizations as time-dependent covariates in panel analyses would be preferred, although doing so would not address the focal issue of calibrating aging effects on changes in cognitive function. On the other hand, there has been considerable variation in how cognitive function has been measured in previous studies of the hazards of hospitalization, and in whether proxy-respondents were considered in those studies [47]. Elsewhere we have shown that relying on proxy-respondents can bias parameter estimates when modelling changes in functional status among older adults (at least for the baseline value of the outcome, affecting stability, regression to the mean, or cumulative advantage or disadvantage interpretations), although excluding them (as we did in this study) introduces selection bias because participants for whom proxies are used generally have poorer health and declining function [48]. Further research is needed to address these discrepant findings.

The second association that we did not find involves either a protective effect against declines in cognitive function linked to physical activity and exercise, or conversely, an increased risk linked to obesity [49-51]. In our study, neither engaging in vigorous activity nor being obese was associated with changes in cognitive function for any of the observed outcomes. This is somewhat puzzling, because previous studies used the MMSE (against which the TICS-7 used in this study has been validated) to assess cognition and comparable self-reports of both physical activity and obesity in similarly aged, large, community-dwelling samples. The third association that we did not find is also somewhat puzzling. We found no association between self-reported smoking status (current or former) with any of the cognitive function outcomes, although there have been numerous empirical reports and reviews documenting an association between cigarette smoking and cognitive decline using comparable measures in community-based samples of older adults [52,53]. The final association that we did not find was between depressive symptoms and cognitive change. This is a bit less puzzling, because the evidence in the literature on this association is rather mixed, with some studies finding it and others not [54-61]. Concern over our failure to find associations between declines in cognitive functioning and physical activity, smoking, and depressive symptoms, however, is tempered by knowing that the USA NIH Conference systematic review rates the quality of the available evidence for all of these associations as "low" [46]. Nonetheless, further research is needed to resolve these seemingly inconsistent findings.

Our study is not without limitations. The most important of these is the reduced sample size due to higher amounts of missing data post-baseline on the TICS-7. Most of that missing data occurred by the first biennial follow-up, and multivariable logistic regression (not shown) revealed that the main risks of this missing data were minority status, low education, and lower baseline TICS-7 scores. Thus, the coefficients shown in Table 5 for race and education are likely underestimates.

## Conclusions

In conclusion, we have shown that declines in cognitive function in the AHEAD cohort were common, with 43.6%, 54.9%, and 52.3% of self-respondents declining on the TICS-7, and immediate and delayed word recall tests, respectively, and only 25.4%, 20.8%, and 22.9% remaining unchanged over an average period of 7.2 years. The main and most consistent risks for declining cognitive function were the baseline values of cognitive function (reflecting substantial regression to the mean), aging (a strong linear pattern of increased decline associated with greater aging, but with diminishing marginal returns), older age at baseline, dying before the end of the study period, lower education, and minority status, with African-Americans declining the most. Unfortunately, no readily modifiable protective factors were identified. Thus, the prospects for the prevention or amelioration of declines in cognitive function are not encouraging given that the world's population continues to age.

## Competing interests

The authors declare that they have no competing interests.

## Authors' contributions

**FDW **conceived of the study, wrote the grant application, designed and conducted the analyses, interpreted the results, and prepared the manuscript. SEB and PAW performed all of the data cleaning and linkage tasks. MPJ, BK, and JH assisted in the design and oversight of the statistical analyses and their interpretation. RBW participated in the conceptualization of the grant application and the overall study design, and provided clinical expertise. All authors participated in numerous meetings to outline, read, critique, revise, re-read, and grant approval the final manuscript. FDW and SEB had full access to all of the data in the study and take responsibility for the integrity of the data and the accuracy of the data analysis. All authors read and approved the final manuscript.

## Pre-publication history

The pre-publication history for this paper can be accessed here:

http://www.biomedcentral.com/1471-2458/11/710/prepub
